# Structural capacity and continuum of snakebite care in the primary health care system in India: a cross-sectional assessment

**DOI:** 10.1186/s12875-023-02109-2

**Published:** 2023-08-11

**Authors:** Soumyadeep Bhaumik, Robyn Norton, Jagnoor Jagnoor

**Affiliations:** 1 The George Institute for Global Health, University of New South Wales, Sydney, Australia; 2 Injury Division, The George Institute for Global Health, New Delhi, India; 3 The George Institute for Global Health, Imperial College, London, UK

**Keywords:** Snakebite, Health systems strengthening, Access to care, Delivery of health care, Primary health care, India, Comprehensive primary health care, Health facility capacity, Continuum of care

## Abstract

**Background:**

In 2019, the World Health Organization, set a target to halve the burden of snakebite, by 2030, and identified ‘health systems strengthening’ as a key pillar of action. In India, the country with most snakebite deaths, the Union Government identified (in September 2022) training of health workers as a priority action area. In this policy context, we provide empirical evidence by analysing the most recent nationwide survey data (District Level Household and Facility Survey − 4), to assess structural capacity and continuum of snakebite care in primary health care system in India.

**Methodology:**

We evaluated structural capacity for snakebite care under six domains: medicines, equipment, infrastructure, human resources, governance and finance, and health management information systems (HMIS). We categorised states (aspirant, performer, front-runner, achiever) based on the proportion of primary health centres (PHC) and community health centres (CHC), attaining highest possible domain score. We assessed continuum of snakebite care, district-wise, under five domains (connectivity to PHC, structural capacity of PHC, referral from PHC to higher facility, structural capacity of CHC, referral from CHC to higher facility) as adequate or not.

**Results:**

No state excelled ( front-runner or achiever) in all six domains of structural capacity in PHCs or CHCs. The broader domains (physical infrastructure, human resources for health, HMIS) were weaker compared to snakebite care medicines in most states/UTs, at both PHC and CHC levels. CHCs faced greater concerns regarding human resources and equipment availability than PHCs in many states. Among PHCs, physical infrastructure and HMIS were aspirational in all 29 assessed states, while medicines, equipment, human resources, and governance and finance were aspirational in 8 (27.6%), 2 (6.9%), 17 (58.6%), and 12 (41.4%) states respectively. For CHCs, physical infrastructure was aspirational in all 30 assessed states/UTs, whereas HMIS, medicines, equipment, human resources, and governance and finance were aspirational in 29 (96.7%), 11 (36.7%), 27 (90%), 26 (86.7%), and 3 (10%) states respectively. No district had adequate continuum of snakebite care in all domains. Except for transport availability from CHC to higher facilities (48% of districts adequate) and transport availability from PHC to higher facilities (11% of districts adequate), fewer than 2% of districts were adequate in all other domains.

**Conclusion:**

Comprehensive strengthening of primary health care, across all domains, and throughout the continuum of care, instead of a piece-meal approach towards health systems strengthening, is necessitated to reduce snakebite burden in India, and possibly other high-burden nations with weak health systems. Health facility surveys are necessitated for this purpose.

**Supplementary Information:**

The online version contains supplementary material available at 10.1186/s12875-023-02109-2.

## Background

Snakebite is a neglected tropical disease (NTD) which primarily affects rural communities in South Asia and sub-Saharan Africa [1, 2]. It is estimated that globally up to 78,600 people died due to snakebite in 2019 [3]. In addition to death, snakebites cause considerable long-term physical disability, has mental health manifestations, and adds to the socio-economic problems, of already deprived communities [2, 4–8]. According to estimates, 65.25% of those who are at risk of being bitten by a snake reside in areas with the lowest access decile to high-quality healthcare, highlighting how unequal access to healthcare and a potential lack of high-quality care can increase vulnerability to severe snakebite envenoming outcomes [9].

In 2019, the World Health Organization (WHO) released a strategy to reduce snakebite related death and disability by 50% by 2030 [10]. One of the four objectives of the WHO strategy is strengthening health systems – with a focus on ensuring time-critical service delivery in primary health care [10, 11]. Snakebite is a medical emergency, and hence care provisioning at the primary healthcare level, which is closer to the geographical site of bite incidents, is essential for reducing mortality and morbidity due to snakebite [1, 10–12]. Snakebite is endemic in rural areas of low- and middle-income countries, where health systems are typically weak. It is acknowledged that health system gaps in terms of availability, access, affordability, and quality are a major barrier in reducing snakebite related death and disability, [10, 12–17] but empirical evidence is lacking. The focus of the current study is India which has the highest number of deaths due to snakebite, and the second highest age-standardised mortality rate globally, next to Somalia [3]. In the current study, we aimed to establish a nation-wide baseline status of health system India, to monitor progress and to identify priority domains for strengthening, by:

1. Assessing structural capacity for snakebite care in the primary health care facilities in the different states of India, and.
2. Analysing district-level adequacy of critical elements for provision of continuum of snakebite care in the primary healthcare system (from village to primary health centre (PHC) and to linked community health centre (CHC)) in India.

For this purpose, we used the District Level Household and Facility Survey (DLHS-4, 2012–2013), the most recent nationwide publicly available dataset which has facility survey data. Despite recent focus on strengthening primary health care in India there is no recent nation-wide facility assessment available (for snakebite or otherwise) [18]. With more than 80% of the global deaths due to snakebite reported in India, the WHO target for 50% reduction in the burden of snakebite by 2030 cannot be attained without reducing the burden in India [3]. Establishing a baseline for health facility capacity for snakebite care, is of current policy relevance in India. The Mission Steering Group, the apex decision making body for strategy and implementation of the National Health Mission, in its 7th meeting held in September 2022 identified inadequate capacity of health workers as a gap and has allocated funding for their training [19]. Our study is conducted under this backdrop, and with a pragmatic stance, with the intention to inform policy formulation through empirical evidence, based on best available data source.

## Methods and analysis

### Context

The primary healthcare systems in India [20] consists of sub-centres (SCs) with linked primary health centres (PHCs). The SCs at the village level focus primarily on preventive and promotive care. A PHC is the first point of medical contact in the public healthcare system, where a medical doctor is available. The PHCs are linked to community health centres (CHCs) which serve as referral points for the PHCs, which in turn are linked to district hospitals (DH) and medical colleges. Overall, a district serves as a self-sufficient unit of the health system wherein all except advanced sub-speciality care is available.

### Data source

The DLHS-4 is a population-linked facility survey conducted by the Ministry of Health and Family Welfare, Government of India. It is primarily aimed to collect district level information on maternal, reproductive and child health and assess progress of related national programs. DLHS-4 is a multi-stage, stratified, probability proportional to size sample with replacement design, cross-sectional, nationally representative survey. In DLHS-4, the primary sampling unit (PSU) in rural areas are villages (as defined by the Census of India 2001 sampling frame) and the PSU for urban areas, Urban Frame Survey (UFS) blocks as per the National Sample Survey Office. The facility component of the survey involved survey of all levels of public health facilities (SC, PHC, CHC, DH) linked to the PSU. The facility survey collected data on infrastructure, staffing, services, and other components related to organisational structure. The data was collected by trained personnel and involved interview of relevant facility personnel, physical observation, and inspection of registers. Further detailed descriptions of the sample methodology and survey process are available in the DLHS website (http://rchiips.org/index.html ).

For this study on snakebite, we use data from the PHC and CHC facility component of DLHS-4 only. We excluded DHs from the analysis because the DLHS facility data on district hospitals did not collect information on availability of snake anti-venom (SAV), a critical drug in the management of snakebite without which assessment of structural capacity or continuum of care is not meaningful. We excluded SCs from the analysis because of the structural design of the public primary health care system, wherein a SC does not have any medical doctor, and thus not a point of contact for snakebite. The training manual for community health workers, who are placed at SC also recommends immediate referral to nearest health facility (PHC or CHC) [21].

### Assessment of structural capacity for acute management of snakebite

**Fig. 1:**
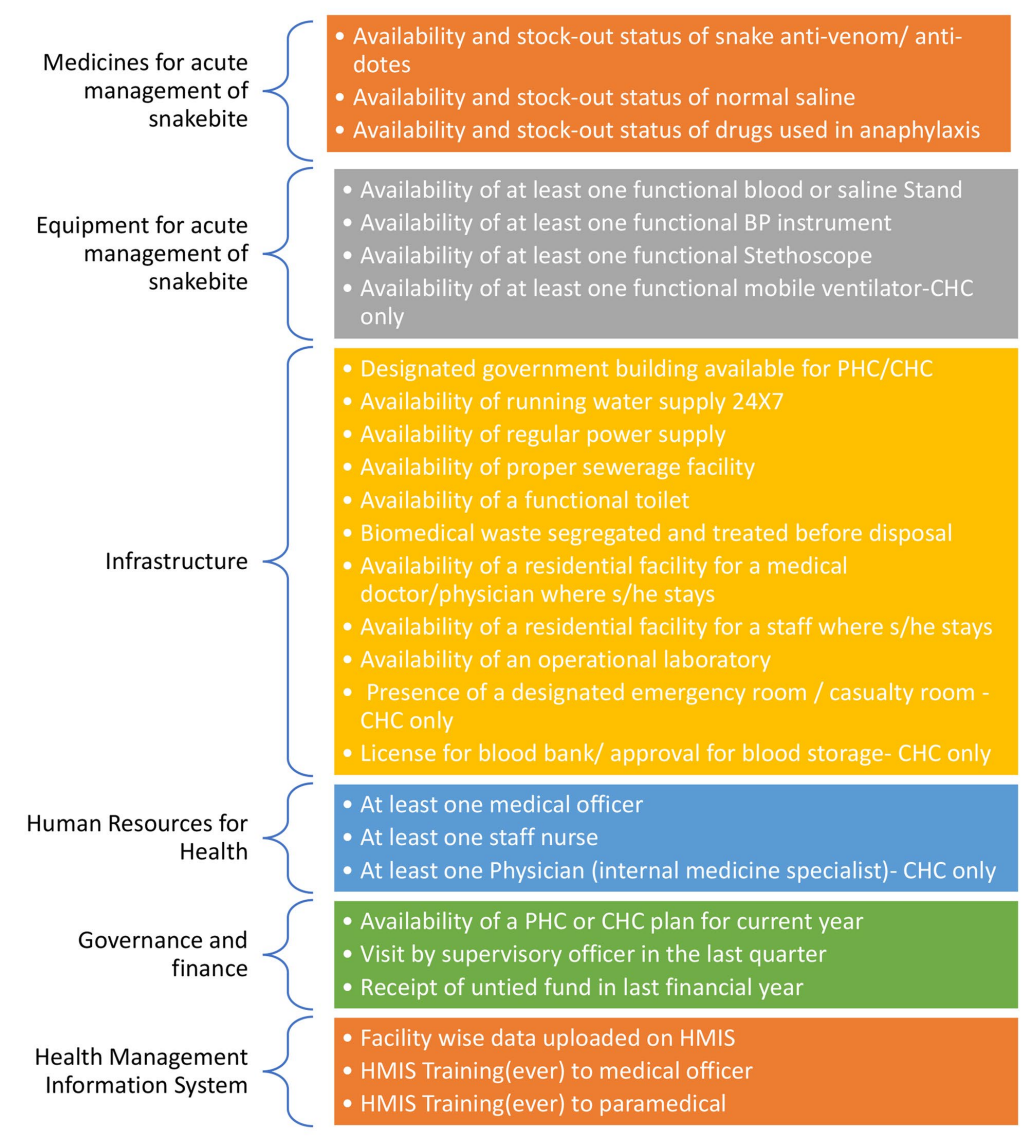
Structural capacity for management of acute snakebite care: domains and indicators

Assessing public health system performance is a complex exercise but essentially has its roots in the Donabedian framework which links structures, processes, outputs and outcomes to understand aspects of quality of care [22]. Turnock and Handler at the Centres for Disease Control and Prevention (CDC), USA [23] first proposed the use of a conceptual framework similar to the Donabedian framework for assessing performance of public health systems. The framework consists of four components (mission, structural capacity, processes, and outcomes) operating in a macro context. Our study pertains to structural capacity alone. We conceptualised structural capacity for snakebite care under six domains (Fig. 1) – two domains specific to snakebite care (medicines for acute management of snakebite, equipment for acute management of snakebite) and four broader ones pertaining to health systems (infrastructure, human resources for health, governance and finance, and health management information systems). The steps for assessing structural capacity involved:

- **Identification of Indicators**: The DLHS-4 survey is not specifically designed to assess any aspect of snakebite care as the focus is primarily on maternal, reproductive and child healthcare. In the absence of any other facility level data on snakebite (at national or state level), the nationally representative DLHS-4 data acts as the best available data source for the purpose. For identifying indicators for the domains of structural capacity specific to snakebite care we mapped the variable in the facility component of DLHS-4 to the national snakebite treatment guidelines [24]. For identifying indicators for the four broader domains of structural capacity, we identified indicators for each of the essential elements of that domain based on the Indian Public Health Standards, [25] and availability of indicators in DLHS-4. Our choice of domains for assessing structural capacity is in alignment with the WHO health systems building blocks and structures and inputs, and the WHO-UNICEF framework for monitoring and measuring primary healthcare [26, 27]. Overall, for the six domains, we had 23 indicators for PHC and 27 indicators for CHC. This includes some 5 composite indicators for PHC (at least one medical doctor, and at least one staff nurse, availability of snake antivenom, availability of normal saline and availability of anaphylaxis drug) and 6 composite indicators for CHC (at least one physician, at least one general duty medical officer, at least one staff nurse, availability of snake antivenom, availability of normal saline and availability of anaphylaxis drug), which we derived from the data. Other indicators were directly available in DLHS-4. In the current public health system in India, management of acute snakebite is a function of doctors and nurses with no formal role to any community health worker. Community health workers are paid to do specific tasks under National Health Mission which does not include any function for snakebite. Detailed descriptions of all the indicators in the six domains for PHC and CHC are available in the Supplementary Appendix 1 and a summary pictorial description is provided in Fig. 1.
- **Normalisation**: We rescaled each indicator as 1 if the structural capacity criterion was positive (for example, if the snake anti-venom was available on the day of the survey and there was no stock-out for more than 10 days during the 30 days preceding the survey it was awarded a score of 1), otherwise we scored it as 0.
- **Weightage**: For each domain, equal weightage was given to each indicator in alignment with the United Nations Sustainable Development Solutions Network methodology [28]. We calculated domain scores for PHC and CHC separately by summing the scores for individual structural capacity element scores in that domain. We did not calculate an overall (or composite) score for structural capacity, but instead present domain-wise scores as overall scores mask domains of strength and weakness, especially in a setting where individual domain scores vary significantly (as is the case in our study). The relative importance of different domains, in a particular context is dependent on multiple factors and can be best decided by state or district level stakeholders. Our approach enables this process (also see strengths and limitations section in Discussion).
- **State domain scores**: We benchmarked the adequacy of structural capacity for domains (separately for CHC and PHC) using cut-off levels, set *a priori*. We classified states into four categories, based on the proportion of health facilities, which could attain the maximal possible score for that domain, as the following:
  - Aspirant: 0 − 49%.
  - Performer: 50–64%.
  - Front-Runner: 65–99%.
  - Achiever: 100%.

The classification benchmark is similar to what National Institution for Transforming India(NITI Aayog), the policy think tank of Government of India uses to classify states as per the sustainable development goal (SDG) India Index. [29, 30]

### Assessment of adequacy of provision of critical elements for continuum of snakebite care

**Fig. 2:**
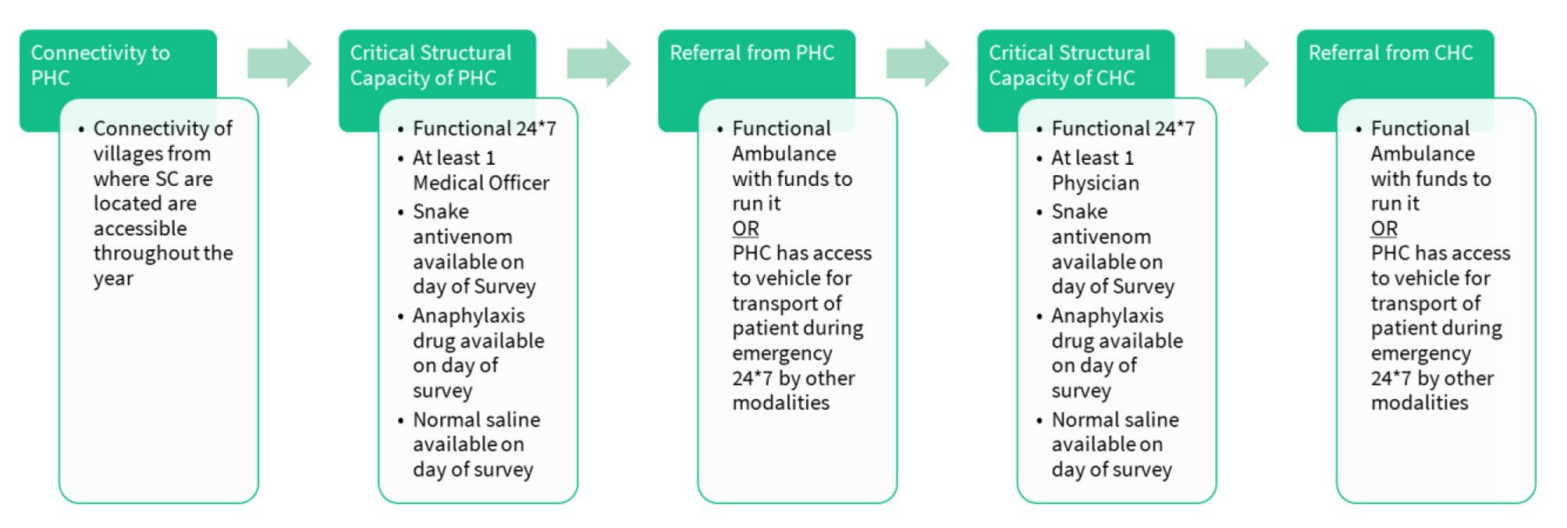
Conceptual framework for provision of critical elements for continuum of snakebite care

Continuum of snakebite care within the public primary health care system in India implies a patient with snakebite would need to reach a PHC, receive care in a PHC, be referred to a CHC, receive care in a CHC, and might be subsequently referred from a CHC to a higher facility. We developed a conceptual model on continuum of snakebite care with five domains, which is reflective of the journey of a person bitten by snake in the public healthcare system. (Fig. 2)

We report descriptive statistics for all analyses. All data analysis was conducted in SPSS.

### Ethics

This study is a secondary analysis of facility level data from a de-identified publicly available national survey. The original DLHS-4 survey received ethics approval from the ethics committee of the International Institute for Population Science (IIPS). Data was requested and obtained from the IIPS Data centre. The data is shared as per a registered access system in accordance with the National Data Sharing and Accessibility Policy of the Government of India [31].

## Results

The DLHS-4 facility survey was conducted nationwide, but we included only those states and union territories (UT) for which data was made publicly available. Data was not available for two states (Gujarat, Jammu and Kashmir – this also includes the current UT of Ladakh which was part of Jammu and Kashmir, when the survey was conducted) and four union territories (Dadra and Nagar Haveli, Daman and Diu, Delhi, and Lakshadweep). Overall, our study included data from involving 8540 PHC’ s from 29 states and 4810 CHCs from 30 states. There was no data from PHC’ s in one state (Chandigarh).

### Structural capacity for acute management of snakebite at PHC level

**Fig. 3:**
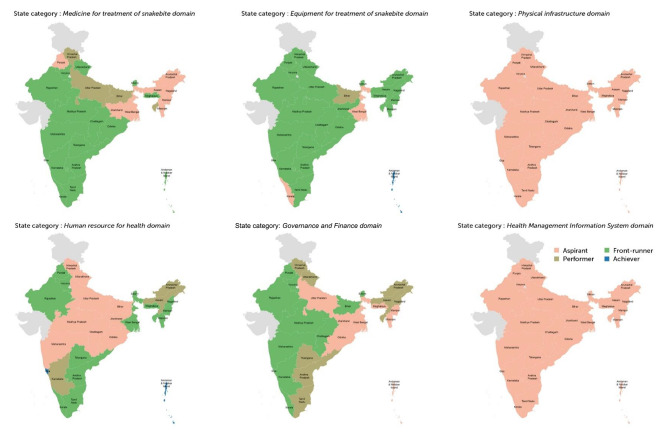
State categorisation of different domains of structural capacity in Primary Health Centres (PHC)

We found that none of the 29 states were front-runners or achievers in all six domains of structural capacity in PHC’ s. The state-level structural capacity for different domains is presented graphically in Fig. 3 and actual scores are presented in Supplementary Appendix 2.

Four of the 29 states (Rajasthan, Haryana, Sikkim, Andhra Pradesh, Goa) were at the front-runner level on four domains (Medicine for treatment of snakebite, Equipment for treatment of snakebite, Human Resources for Health, Governance and Finance), which was the highest level attained. Summary statistics of the structural capacity of PHC in states/UT’s for snakebite care in different domains are:

1. Medicine for treatment of snakebite domain: 17 states /UTs were front-runners, four were performers and eight aspirants.
2. Equipment for treatment of snakebite domain: One UT (Andaman and Nicobar Island) was an achiever, 25 states were front-runners, one was a performer and two aspirants.
3. Physical infrastructure domain: 29 states /UTs were aspirants.
4. Human Resources Domain: 17 states/UTs were front-runners, three were performers and nine were aspirants.
5. Governance and Finance domain: 12 states/UTs were front-runners, eight were performers and nine were aspirants.
6. Health Management Information Systems domain: 29 states /UTs were aspirants.

### Structural capacity for acute management of snakebite at CHC level

**Fig. 4:**
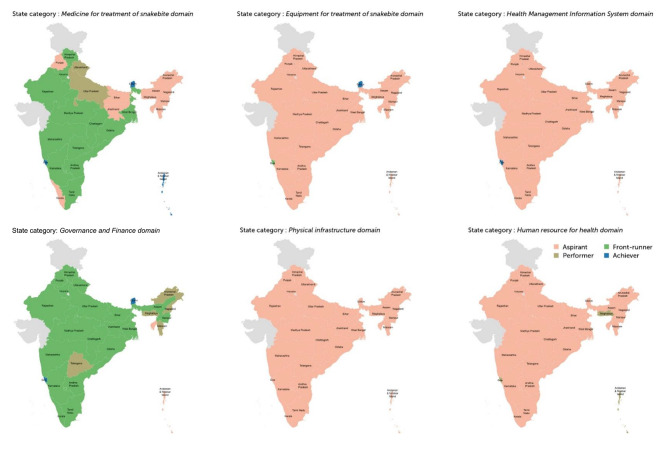
State categorisation of different domains of structural capacity in Community Health Centres (CHC)

Overall, we found that none of the 30 states were front-runners or achievers in all six domains of structural capacity in CHCs. The state-level structural capacity for different domains is presented graphically in Fig. 4 and actual scores are presented in Supplementary Appendix 3.

Sikkim was an achiever in three domains (Medicine for treatment of snakebite, Equipment for treatment of snakebite, Governance and finance) and Goa was an achiever in two domains (Medicine for treatment of snakebite, Governance and finance) and front-runner in one domain (Equipment for treatment of snakebite). These two states attained the highest levels. The structural capacity of CHCs in states/UTs for snakebite care in different domains are:

1. Medicine for treatment of snakebite domain: Three states /UTs are achievers (Sikkim, Goa, Andaman and Nicobar Islands), 13 states /UTs were front-runners, three were performers and 11 aspirants.
2. Equipment for treatment of snakebite domain: Two states /UTs are achievers (Chandigarh and Sikkim), one is a front-runner, and 27 are aspirants.
3. Physical infrastructure domain: 30 states /UTs were aspirants.
4. Human Resources Domain: Four were performers and 26 were aspirants.
5. Governance and Finance domain: Two states were achievers (Sikkim and Goa), 19 states/UTs were front-runners, six were performers and three were aspirants.
6. Health Management Information Systems domain: 29 states /UTs were aspirants.

### Adequacy of continuum of snakebite care

**Fig. 5:**
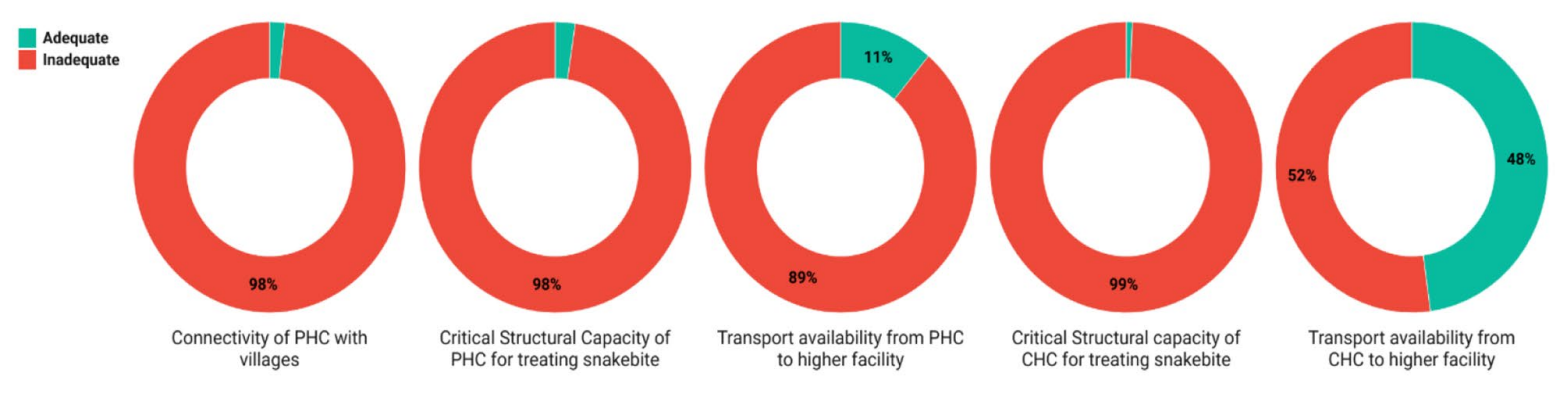
Proportion of districts (nation-wide) which had adequacy in domains related to continuum of snakebite care

Overall, we found that none of the districts in any of the 30 states had adequate provision of continuum of snakebite care in the public primary health system. The overall nation-wide summary of district-level domains which constituted continuum of snakebite care is summarised below and in Fig. 5 (details, including with names of districts in each state is included in the Supplementary Appendix 4):

1. accessibility of PHC throughout the year: was adequate in ten districts in three states,
2. structural capacity of PHC to manage acute snakebite care: was adequate in 13 districts in six states,
3. availability of functional transport system for referral from PHC to higher centre: was adequate in 61 districts in 15 states,
4. structural capacity of CHC to manage acute snakebite care CHC: was adequate in four districts in three states,
5. availability of functional transport system for referral from CHC to higher centre was adequate in 262 districts in 29 states.

West Bengal was the only state where all districts were found to be inadequate for all domains which constituted continuum of snakebite care. In 10 states (Telangana, Goa, Karnataka, Andhra Pradesh, Tripura, Manipur, Nagaland, Sikkim, Odisha, Uttar Pradesh), all districts were found to be inadequate for four of the five domains which constitute continuum of snakebite care.

## Discussion

### Summary of key results

This study presents state-level data on structural capacity and district-level data on adequacy of continuum of snakebite care in India – the first such study globally. We found that broader health systems domains (physical infrastructure, human resources for health, health management for information systems) are structurally weaker than the domain of medicines required for treatment of snakebite (snake anti-venom, anaphylaxis management drugs and normal saline) for almost all states, both at PHC and CHC level, although they were also not optimal. Availability of human resources for health and equipment was of greater concern in CHC than at PHC in many states. The continuity of care analysis affirms the above finding. The lack of accessibility of PHC throughout the year and the lack of effective referral linkage from PHC to higher centre, are additional critical gaps identified through the continuum of care analysis. Critical structural capacity at PHC and CHC, which is the minimum capacity required for delivery of snakebite care was inadequate in almost all districts of India. There was, however, inter-state and intra-state variation.

### Study findings within the context of what is previously known

The results of the study are based on the most recent nation-wide data that is available publicly, which was collected in 2012–2013. As such, the study provides insight on priority areas of focus for comprehensive health systems strengthening and establishes a baseline for monitoring progress. The results of the study should also be seen considering other data, available over time, for some indicators. The Rural Health Statistics 2012, which correspond to the period when the DLHS-4 was conducted, reported a shortfall of 10.3% for medical doctors at PHC level and 79.6% for physicians at CHC level [32] The shortfall reported in Rural Health Statistics of 2021, is 4.3% for medical doctors in PHC and 82.2% for physicians in CHC [33]. This indicates discordance between administrative data (which reports data ‘on paper’ basis) with survey data. The administrative data shows improvement in medical doctor at PHC level and deterioration at CHC level in the past decade.

There has been broader economic development, much of which might impact the infrastructure domain of structural capacity. As for example, between 2012 and 2020, access to electrification (%age of population) has increased from 79.9 to 99.0% in India [34]. However, the Annual Health Statistics, as reported in 31st March 2021, show 4.8% of rural PHCs still have no electric supply at all [33].It is known that poor availability of electricity in PHC is disproportionately associated with access and quality of maternal care in India [35].A quasi-experimental evaluation of the *Pradhan Mantri Gram Sadak Yojana*(which is tasked with constructing all-weather roads in all eligible unconnected rural habitations) found that between 2010 and 2015 the program led to a statistically significant increase in the probability of a woman being delivered in a health facility, but there was no evidence of decreased neonatal mortality rate or post-partum complications [36]. This indicates the need for focussing on quality of care. The same principle would hold true for health systems strengthening for snakebite care. Previous analysis of capacity for health for intrapartum care and cervical cancer, in India, have also identified infrastructure and staffing as critical gaps in continuum of care [37, 38].

### Strengths and limitations

The DLHS-4 facility survey is primarily geared towards reproductive, maternal and child health. Our analysis is focussed on assessment of structural capacity on snakebite care. The elements analysed are only those that are incidentally captured in the survey. The study results should be seen in this light, implying a more comprehensive assessment of health facilities, might demonstrate an even worse result.

Overall, this study provides a baseline, for future assessments. It is also noteworthy, that the results of the study are indicative of only structural capacity and does not provide any information on functional capacity or quality of care. There is also a need to understand and address the “intangible software” of health systems, i.e., the “ideas, norms, values and issues of power or trust that affect the performance of health systems” [39].

We did not calculate any overall score for structural capacity or continuum of care, and instead provided domain wise information to enable better visualisation of systems gaps and key areas of improvement. An overall scoring obliterates identification of bottle necks especially in the scenario when individual domain scores vary tremendously, as in our case. It is important to note that our domains pertain to structural capacity alone and not reflective of functional capacity. This is particularly relevant to the domain of governance and financing where the importance of intangible software (i.e., ideas, norms, values, attitudes, and relationships in the public health system [39]) is of critical importance. The results pertaining to governance and financing (green in most states) should be interpreted in this light. Another limitation also pertains to the reliability and specificity of the few questions related to infrastructure in DLHS-4 itself. Instead of subjectively asking respondents whether the power supply was regular, or sewerage facility was proper, or whether the toilet was proper and in-use, future iterations of DLHS should use more objective measures. For example, the number of hours of power supply in the last 24 h and structure observation on sewerage and toilet would enhance data quality. Elements form the questionnaire of the National Annual Rural Sanitation Survey [40] might be comprehensive for assessment of sewerage and sanitation in health facilities assessments in the future.

### Implications for policy and practice

The roadmap by the Indian Council of Medical Research - National Task Force for Research on Snakebite focussed on development of rapid diagnostics kits and snake antivenom, guideline dissemination, legislative changes, awareness, and media outreach [41]. The Mission Steering Group, the apex decision making body for strategy development as well as implementation of the National Health Mission, in its 7th meeting held in September 2022 prioritised community awareness and capacity building of health workers for addressing snakebite [19]. However, based on our findings we contend that the piece-meal approach will not lead to the adequate health system strengthening for addressing the snakebite burden. A comprehensive approach is required to deliver on the continuum of primary health care for desired reduction in the snakebite burden. In policy terms, the Union Government of India should also consider commissioning a nationwide health facility assessment in high snakebite burden states.

Our analysis and available information indicate that even a decade back, the weakest elements of structural capacity were infrastructure, equipment, availability of human resources for health and health management information systems. The availability of ventilators is a critical infrastructure with respect to snakebite, is a key gap.

We recommend that states should conduct rapid health facility assessments using a systems approach, with snakebite care specific indicators integrated within the exercise. Concurrent use of a continuous structure and process improvement models at district level with special attention to snakebite care will enable better contextual understanding for improvement of both quality and access.

The dominant focus of global funders and researchers is to develop newer or region specific snake anti-venoms [42]. With up to 64,100 Indians dying from snakebite every year in India, [3] re-orienting investments for snakebite towards comprehensive strengthening of primary healthcare (along with prevention), has the potential to save many lives in the immediate and medium term, and guarantee delivery of newer and improved therapeutic products, as and when they become available in the distant future.

Our data is from India, however similar scenario might be expected in other high-burden nations in Asia, and Africa, which are known to have weak health systems [43, 44]. In general, there is need for health facility assessments with focus on snakebite. Currently there is no facility checklist or standard for snakebite care in India or globally. Development of a comprehensive health facility checklist and facility level standards of snakebite care (best not as standalone but integrated within existing ones or multi-disease in nature), will enable strengthening of the public primary health care system, leading to decreasing the burden of snakebite. Development of contextually relevant facility standards and checklist will enable more comprehensive assessment of capacity of snakebite care in high-burden nations.

The NITI Aayog Health Index uses similar methodology to categorise states for health systems functioning [30]. The index however is derived from indicators pertaining mostly to reproductive, maternal, and child health, tuberculosis, and HIV. There are no indicators specific to snakebite, or acute medical emergencies, for other conditions. Our data shows, that even high-performing states (as per NITI Aayog) did not have good scores for structural capacity for snakebite care. Integration of indicators related to care for snakebite, a neglected tropical disease, within the NITI Aayog Health Index can make the index more equity sensitive. Such an integration aligns with the Union Government commitment to “leave no one behind by making the index more comprehensive and realistic, and act as a nudge for states to address snakebite.

## Conclusion

Comprehensive health system strengthening, focussing on all health systems blocks, and throughout the continuum of snakebite care in the primary health care system, instead of a piece-meal approach towards health systems strengthening, is critical for reducing the burden of snakebite in India, and potentially in other high-burden nations with weak health systems. For this purpose, nationwide facility surveys are necessitated. In India, we also suggest the addition of indicators related to snakebite care in future iterations of the NITI Aayog Health Index. This would make the index more comprehensive, realistic and equity focussed.

### Electronic supplementary material

Below is the link to the electronic supplementary material.

**Supplementary Material 1**

**Supplementary Material 2**

**Supplementary Material 3**

**Supplementary Material 4**

## Data Availability

The data underlying the results presented in the study, available as supplementary appendices. All data underlying the results is from a freely available public data set of the District Level Household and Facility Survey (DLHS-4). The data is available for academic research on request to the Data Centre of the International Institute for Population Sciences (IIPS), an autonomous institute under the aegis of Ministry of Health and Family Welfare, Government of India. The datasets underlying this article is available from the following source: https://www.iipsindia.ac.in/content/data-request.
