# Supplementary material for: Structural capacity and continuum of snakebite care in the primary health care system in India: a cross-sectional assessment
Source: BMC Prim Care. 2023 Aug 11;24:160. doi: 10.1186/s12875-023-02109-2 (PMC10416377; doi:10.1186/s12875-023-02109-2)
Supplement: Supplementary file 1 — Supplementary Material 1 [file 12875_2023_2109_MOESM1_ESM.docx]

Supplementary appendix 1 : structural capacity of snakebite care

Contents

[Variable definitions and mapping for structural capacity in PHC 2](#_Toc117464559)

[Variables for structural capacity in CHC 4](#_Toc117464560)

# Variable definitions and mapping in facility DLHS-4 Questionnaire for structural capacity in PHC

| **Sl no.** | **Question DLHS** | **Variable number in DLHS-4 questionnaire** |
| --- | --- | --- |
| **PHYSICAL INFRASTRUCTURE** | | |
|  | Designated government building available for PHC | 4.2 |
|  | Running water supply for 24X7 | 4.9 |
|  | Regular power supply | 4.10 |
|  | Proper sewerage facility available | 4.12 |
|  | Toilet available and in use | 4.14a |
|  | Biomedical waste segregated and treated before disposal | 4.23 |
|  | Residential facility available for doctor (available AND staying) | 4.26a-C |
|  | Residential facility available for nurse (available AND staying) | +4.26d-C |
|  | Operational laboratory | 1.12 |
| **HUMAN RESOURCES FOR HEALTH** | | |
|  | At least one medical doctor | 21a OR 21b OR 22a OR 22b |
|  | At least one staff nurse | 2.5a OR 2.5b |
| **GOVERNANCE AND FINANCE** | | |
|  | PHC plan | 8.29 |
|  | Supervisory officer visited last month | 8.35 |
|  | Received untied fund in last FY | 8.43 |
| **HEALTH MANAGEMENT INFORMATION SYSTEMS** | | |
|  | Facility wise data uploaded on HMIS | 9.10 |
|  | Training on HMIS BY MO (EVER) | 3.12 b ever |
|  | Training on HMIS BY paramedical (EVER) | 3.18 ever |
| **MEDICINE FOR ACUTE SNAKEBITE TREATMENT** | | |
|  | Availability of snake anti-venom/anti-dotes  AND | 6.11 a |
|  | NO Stock-out of snake anti-venom/anti-dotes | 6.11b |
|  | Snake antivenom availability | 6.11a+6.11b |
|  | Availability of Normal saline  AND | 6.14b1 |
|  | NO Stock-out of Normal saline | 6.14b2 |
|  | Normal saline availability | 6.14b1+6.14b2 |
|  | Availability of anti-allergic and drugs used in anaphylaxis  AND | 6.1a |
|  | NO Stock-out of anti-allergic and drugs used in anaphylaxis | 6.1b |
|  | Anaphylaxis drug availability | 6.1a+6.1b |
| **EQUIPMENT FOR SNAKEBITE CARE** | | |
|  | Available and functional Blood/Saline Stand | 4.43 |
|  | Available and functional BP instrument | 4.52 |
|  | Available and functional Stethoscope | 4.53 |

# Variable definitions and mapping in facility DLHS-4 Questionnaire for structural capacity in CHC

#

| Sl no. | **Question DLHS** | **Variable number in DLHS-4 questionnaire** |
| --- | --- | --- |
| **PHYSICAL INFRASTRUCTURE** | | |
|  | Designated government building available for CHC | 5.2 |
|  | Running water supply 24*7 | 5.9 |
|  | Regular power supply | 5.12 |
|  | Proper sewerage facility available | 5.16 |
|  | Toilet available and in use | 5.18a |
|  | Biomedical waste segregated and treated before disposal | 5.25 |
|  | Residential facility available for Physician (available AND staying) | 5.32 |
|  | Residential facility available for staff nurse (available AND staying) | 5.36 |
|  | Operational laboratory | 5.59 |
|  | Designated emergency room / casualty room available in CHC | 5.70 |
|  | License for blood bank/ approval for blood storage | 1.8 |
| **HUMAN RESOURCES FOR HEALTH** | | |
|  | At least one Physician | 2.2a OR 2.2b |
|  | At least one Medical Officer (General Deuty) | 2,9a OR 2.9b |
|  | At least one staff nurse | 2.13a OR 2.13b |
| **GOVERNANCE AND FINANCE** | | |
|  | CHC plan | 11.1 |
|  | Supervisory officer visited last quarter | 11.3 |
|  | Received untied fund in last FY | 11.8 |
| **HEALTH MANAGEMENT INFORMATION SYSTEMS** | | |
|  | Facility wise data uploaded on HMIS | 12.11 |
|  | Training on HMIS BY MO (EVER) | 3.20b ever |
|  | Training on HMIS BY paramedical (EVER) | 3.29bever |
| **MEDICINE FOR ACUTE SNAKEBITE TREATMENT** | | |
|  | Availability of anti-dotes / snake anti-venom  AND | 811 a |
|  | NO Stock-out of anti-dotes / snake anti-venom | 811b |
|  | Snake antivenom availability | 811a+811b |
|  | Availability of Normal saline  AND | 8.14b1 |
|  | NO Stock-out of Normal saline | 8.14b2 |
|  | Normal saline availability | 8.14b1+8.14b2 |
|  | Availability of anti-allergic and drugs used in anaphylaxis  AND | 81a |
|  | NO Stock-out of anti-allergic and drugs used in anaphylaxis | 81b |
|  | Anaphylaxis drug availability | 81a+81b |
| **EQUIPMENT FOR SNAKEBITE CARE** | | |
|  | Available and functional Blood/Saline Stand | 5.86c |
|  | Available and functional BP instrument | 5.86j |
|  | Available and functional Stethoscope | 5.86k |
|  | Available and functional mobile ventilator | 7.3 |
