# Supplementary material for: Structural capacity and continuum of snakebite care in the primary health care system in India: a cross-sectional assessment
Source: BMC Prim Care. 2023 Aug 11;24:160. doi: 10.1186/s12875-023-02109-2 (PMC10416377; doi:10.1186/s12875-023-02109-2)
Supplement: Supplementary file 2 — Supplementary Material 2 [file 12875_2023_2109_MOESM2_ESM.docx]

Supplementary appendix 2: Proportion of PHCs having highest score in different domains of structural capacity for snakebite care in India

| **Name of State** | **Medicine for treatment of snakebite** | **Equipment for treatment of snakebite** | **Physical Infrastructure** | **Human Resources for Health** | **Governance and Finance** | **Health Management Information System** |
| --- | --- | --- | --- | --- | --- | --- |
| **Uttarakhand** | 72.6% | 86.9% | 7.1% | 46.4% | 55.4% | 0.0% |
| **Rajasthan** | 79.5% | 81.4% | 3.3% | 71.1% | 72.3% | 1.3% |
| **Uttar Pradesh** | 60.3% | 70.5% | 0.1% | 4.8% | 23.5% | 2.0% |
| **Bihar** | 52.7% | 60.7% | 2.1% | 38.6% | 72.7% | 5.4% |
| **Assam** | 47.7% | 93.1% | 9.6% | 61.6% | 53.6% | 0.7% |
| **Jharkhand** | 37.0% | 69.1% | 0.0% | 14.5% | 34.5% | 0.0% |
| **Odisha** | 77.6% | 78.9% | 0.0% | 8.1% | 31.8% | 0.4% |
| **Chhattisgarh** | 77.8% | 91.7% | 2.0% | 22.2% | 66.3% | 0.0% |
| **Madhya Pradesh** | 84.1% | 83.0% | 1.6% | 12.5% | 72.8% | 0.0% |
| **Himachal Pradesh** | 58.8% | 73.9% | 1.3% | 28.1% | 61.4% | 18.2% |
| **Punjab** | 26.5% | 81.5% | 0.6% | 66.0% | 72.8% | 0.0% |
| **Haryana** | 81.7% | 91.5% | 1.6% | 78.9% | 85.4% | 8.3% |
| **Sikkim** | 75.0% | 91.7% | 29.2% | 66.7% | 66.7% | 0.0% |
| **Arunachal Pradesh** | 17.1% | 81.7% | 2.4% | 50.0% | 50.0% | 0.0% |
| **Nagaland** | 28.4% | 86.4% | 3.4% | 73.9% | 51.1% | 15.0% |
| **Manipur** | 23.7% | 81.4% | 0.0% | 81.4% | 37.3% | 0.0% |
| **Mizoram** | 42.9% | 97.6% | 31.0% | 92.9% | 47.6% | 16.7% |
| **Tripura** | 63.6% | 90.9% | 11.4% | 93.2% | 62.8% | 12.9% |
| **Meghalaya** | 92.0% | 97.3% | 16.0% | 73.3% | 9.3% | 0.0% |
| **West Bengal** | 12.7% | 39.9% | 4.8% | 75.9% | 36.0% | 0.0% |
| **Maharashtra** | 96.5% | 97.0% | 25.4% | 28.4% | 91.4% | 2.6% |
| **Andhra Pradesh** | 92.5% | 94.2% | 0.3% | 86.7% | 64.5% | 4.2% |
| **Karnataka** | 83.7% | 95.7% | 1.3% | 56.0% | 86.1% | 2.3% |
| **Goa** | 76.5% | 88.2% | 0.0% | 100.0% | 76.5% | 0.0% |
| **Kerala** | 76.2% | 44.2% | 1.1% | 77.9% | 76.1% | 5.3% |
| **Tamil Nadu** | 86.8% | 93.9% | 2.8% | 73.6% | 62.9% | 0.0% |
| **Puducherry** | 77.3% | 95.7% | 13.0% | 69.6% | 36.4% | 0.0% |
| **Andaman and Nicobar Island** | 94.4% | 100.0% | 16.7% | 100.0% | 33.3% | 0.0% |
| **Telengana** | 94.9% | 94.9% | 2.0% | 90.9% | 64.0% | 5.4% |
