# Supplementary material for: Structural capacity and continuum of snakebite care in the primary health care system in India: a cross-sectional assessment
Source: BMC Prim Care. 2023 Aug 11;24:160. doi: 10.1186/s12875-023-02109-2 (PMC10416377; doi:10.1186/s12875-023-02109-2)
Supplement: Supplementary file 3 — Supplementary Material 3 [file 12875_2023_2109_MOESM3_ESM.docx]

Supplementary appendix 3: Proportion of CHCs having highest score in different domains of structural capacity for snakebite care in India

| **Name of State** | **Medicine for acute snakebite Treatment** | **Equipment for Acute Snakebite treatment** | **Physical Infrastructure** | **Human Resources for Health** | **Governance and Finance** | **Health Management Information System** |
| --- | --- | --- | --- | --- | --- | --- |
| **Uttarakhand** | 55.0% | 10.0% | 0.0% | 13.3% | 68.3% | 0.0% |
| **Rajasthan** | 75.2% | 12.7% | 3.8% | 24.1% | 86.4% | 0.7% |
| **Uttar Pradesh** | 62.2% | 3.2% | 0.1% | 16.1% | 77.7% | 0.8% |
| **Bihar** | 48.5% | 20.6% | 1.5% | 27.9% | 75.0% | 0.0% |
| **Assam** | 19.7% | 4.7% | 0.9% | 14.5% | 80.4% | 0.0% |
| **Jharkhand** | 36.3% | 7.2% | 0.0% | 3.9% | 77.2% | 0.0% |
| **Odisha –** | 78.2% | 5.6% | 0.3% | 10.3% | 73.1% | 0.0% |
| **Chhattisgarh** | 76.1% | 4.3% | 0.0% | 10.5% | 79.0% | 0.0% |
| **Madhya Pradesh** | 88.4% | 6.8% | 2.2% | 10.6% | 88.3% | 0.0% |
| **Himachal Pradesh** | 71.4% | 11.7% | 1.3% | 7.8% | 72.4% | 14.9% |
| **Punjab** | 41.2% | 28.3% | 0.8% | 33.3% | 79.2% | 7.1% |
| **Chandigarh** | 50.0% | 100.0% | 0.0% | 50.0% | 50.0% | 0.0%** |
| **Haryana** | 85.7% | 7.5% | 0.9% | 2.8% | 89.5% | 0.0% |
| **Sikkim** | 100.0% | 100.0% | 0.0% | 0.0% | 100.0% | 0.0% |
| **Arunachal Pradesh** | 22.6% | 5.7% | 0.0% | 5.7% | 56.6% | 0.0% |
| **Nagaland** | 5.0% | 19.0% | 0.0% | 23.8% | 61.9% | 40.0% |
| **Manipur** | 0.0% | 6.3% | 0.0% | 6.3% | 68.8% | 8.3% |
| **Mizoram** | 36.4% | 27.3% | 0.0% | 18.2% | 63.6% | 12.5% |
| **Tripura** | 28.6% | 0.0% | 0.0% | 0.0% | 45.5% | 0.0% |
| **Meghalaya** | 0.0% | 21.4% | 0.0% | 57.1% | 64.3% | 0.0% |
| **West Bengal** | 82.8% | 4.6% | 0.3% | 7.4% | 67.4% | 0.0% |
| **Maharashtra** | 93.2% | 32.9% | 3.2% | 12.9% | 89.9% | 4.5% |
| **Andhra Pradesh** | 85.0% | 3.8% | 1.9% | 6.4% | 81.3% | 0.0% |
| **Karnataka** | 85.2% | 15.1% | 0.5% | 8.6% | 88.2% | 4.0% |
| **Goa** | 100.0% | 75.0% | 0.0% | 50.0% | 100.0% | 0.0%** |
| **Kerala** | 42.7% | 29.9% | 14.6% | 37.2% | 88.2% | 35.7% |
| **Tamil Nadu** | 89.7% | 19.9% | 1.2% | 31.7% | 87.2% | 0.9% |
| **Puducherry** | 85.7% | 71.4% | 0.0% | 28.6% | 42.9% | 0.0% |
| **Andaman and Nicobar** | 100.0% | 0.0% | 0.0% | 50.0% | 25.0% | 0.0% |
| **Telengana** | 81.6% | 13.6% | 2.3% | 9.1% | 64.3% | 16.4% |
