# Supplementary material for: Structural capacity and continuum of snakebite care in the primary health care system in India: a cross-sectional assessment
Source: BMC Prim Care. 2023 Aug 11;24:160. doi: 10.1186/s12875-023-02109-2 (PMC10416377; doi:10.1186/s12875-023-02109-2)
Supplement: Supplementary file 4 — Supplementary Material 4 [file 12875_2023_2109_MOESM4_ESM.docx]

Supplementary Appendix 4: State-wise listing of Indian districts with adequate continuum of snakebite care under different domains

| **Name of State** | **Connectivity of PHC with villages** | **Critical Structural Capacity of PHC for treating snakebite** | **Transport availability from PHC to higher facility** | **Critical Structural capacity of CHC for treating snakebite** | **Transport availability from CHC to higher facility** |
| --- | --- | --- | --- | --- | --- |
| **Uttarakhand** | 1. Rudryaprayag | 1. Uttarkashi 2. Rurdraprayag 3. Champawat | 1. Uttarkashi 2. Rurdraprayag 3. Garhwal 4. Nainital | 1. Champawat | 1. Uttarkashi 2. Chamoli 3. Rudraprayag 4. Tehri Garhwal 5. Dehradun 6. Garhwal 7. Pithoragarh 8. Bageshwar 9. Champawat 10. Udham Singh Nagar |
| **Rajasthan-** | 0 | 0 | 1. Sirohi | 0 | 1. Ganganagar 2. Hamumagarh 3. Bikaner 4. Churu 5. Karauli 6. Sawai Madhopur 7. Dausa 8. Jaipur 9. Nagaur 10. Sirohi 11. Pali 12. Ajmer 13. Tonk 14. Bundi 15. Bhilwara |
| **Uttar Pradesh** | 0 | 0 | 0 | 0 | 1. Saharanpur 2. Jyotiba Phule Nagar 3. Aligarh 4. Hathras 5. Firozabad 6. Etah 7. Mainpuri 8. Budaun 9. Pilibhit 10. Shahjahanpur 11. Kheri 12. Lucknow 13. Rae Bareli 14. Etawah 15. Auraiya 16. Jhansi 17. Lalitpur 18. Chitrakoot 19. Barabanki 20. Basti 21. Azamgarh 22. Sant Ravidas Nadar Bhadohi 23. Sonbhadra |
| **Bihar** | 0 | 0 | 0 | 1. Muzzafarpur 2. Lakhisarai | 1. Pashchim Champaran 2. Purba Champaran 3. Sheohar 4. Supaul 5. Araria 6. Kishanganj 7. Purnia 8. Katihar 9. Madhepura 10. Saharsa 11. Muzaffarpur 12. Vaishali 13. Samastipur 14. Khagaria 15. Munger 16. Lakhisarai 17. Sheikhpura 18. Patna 19. Bhojpur 20. Rohtas 21. Jehanabad 22. Aurangabad |
| **Assam** | 1. Hailakandi | 0 | 1. Hailakandi | 0 | 1. Kokrajhar 2. Goalpara 3. Nagaon 4. Golaghat 5. Karbi Anglong 6. North Cachar Hills 7. Hailakandi |
| **Jharkhand** | 1. Kodarma 2. Purbi Singhbhum | 0 | 1. Kodarma 2. Giridh 3. Godda | 0 | 1. Godda 2. Bokaro 3. Purbi Singhbhum |
| **Odisha** | 0 | 0 | 0 | 0 | 1. Kendrapara 2. Nuapada 3. Rayagada 4. Koraput 5. Malkangiri |
| **Chhattisgarh** | 0 | 0 | 1. Kawardha 2. Rajnandgaon | 0 | 1. Koriya 2. Jashpur 3. Bilaspur 4. Kawardha 5. Rajnandgaon 6. Raipur 7. Mahasamund 8. Dhamtari 9. Bastar 10. Dantewada |
| **Madhya Pradesh- high burden** | 0 | 0 | 1. Panna 2. Umaria 3. Shahdol 4. Ujjain 5. Jhabua 6. Seoni | 0 | 1. Sheopur 2. Guna 3. Tikamgarh 4. Chhatarpur 5. Panna 6. Damoh 7. Umaria 8. Mandsaur 9. Ratlam 10. Ujjain 11. Dewas 12. Jhabua 13. Dhar 14. Indore 15. Barwani 16. East Nimar 17. Rajgarh 18. Bhopal 19. Raisen 20. Betul 21. Katni 22. Dindori 23. Seoni 24. Balaghat |
| **Himachal Pradesh** | 0 | 0 | 1. Kullu | 0 | 1. Chambra 2. Kullu 3. Hamirpur 4. Bilaspur 5. Sirmaur |
| **Punjab** | 0 | 0 | 1. Rupnagar 2. Fatehgarh Sahib | 0 | 1. Gurdaspur 2. Amritsar 3. Kapurthala 4. Shahid Bhagat Singh Nagar 5. Rupnagar 6. Fatehgarh Sahib 7. Ludhiana 8. Moga 9. Faridkot 10. Mansa 11. SAS Nagar 12. Barnala 13. Taran Taran |
| **Chandigarh** | NA | NA | NA | 0 | 1. Chandigarh |
| **Haryana** | 0 | 1. Panchkula | 1. Kurukshetra 2. Karnal 3. Jind 4. Bhiwani 5. Rohtak | 0 | 1. Panchkula 2. Ambala 3. Yamunanagar 4. Kurukshetra 5. Kaithal 6. Karnal 7. Panipath 8. Jind 9. Fatehabad 10. Hisar 11. Rohtak 12. Faridabad 13. Mewat 14. Palwal |
| **Sikkim** | 0 | 0 | 0 | 0 | 1. South Sikkim |
| **Arunachal Pradesh** | 1. Lower Subansiri 2. Upper Siang 3. Dibang Valley | 0 | 1. Lohit | 0 | 1. Tawang 2. West Kameng 3. East Kameng 4. Papumpare 5. Changlang 6. Anjaw |
| **Nagaland** | 0 | 0 | 0 | 0 | 1. Mon 2. Tuensang 3. Mokokchung 4. Zunheboto 5. Dimapur 6. Kohima 7. Phek 8. Paren |
| **Manipur** | 0 | 0 | 0 | 0 | 1. Tamenglong 2. Imphal West |
| **Mizoram** | 0 | 0 | 0 | 1. Kolasib | 1. Kolasib 2. Aizawl 3. Champhai 4. Serchhip 5. Lunglei 6. Lawngtlai |
| **Tripura** | 0 | 0 | 0 | 0 | 1. Dhalai |
| **Meghalaya** | 0 | 0 | 1. West Garo Hills 2. East Garo Hills 3. South Garo Hills 4. West Khasi Hills 5. Ri Bhoi | 0 | 1. West Garo Hills 2. South Garo Hills 3. West Khasi Hills 4. East Khasi Hills 5. Jaintia Hills |
| **West Bengal** | 0 | 0 | 0 | 0 | 0 |
| **Maharashtra** | **0** | 1. Nandurbar 2. Dhule 3. Amrawati 4. Jalna 5. Satara | 1. Nandurbar 2. Dhule 3. Jalgaon 4. Buldana 5. Washim 6. Amrawati 7. Nagpur 8. Bhandara 9. Gondiya 10. Gadchiroli 11. Chandrapur 12. Yavatmal 13. Jalna 14. Aurangabad 15. Nashik 16. Thane 17. Pune 18. Ahmadnagar 19. Latur 20. Osmanabad 21. Solapur 22. Satara 23. Ratnagiri 24. Sindhudurg 25. Kolhapur | 0 | 1. Nandurbar 2. Dhule 3. Jalgaon 4. Buldana 5. Akola 6. Washim 7. Amrawati 8. Wardha 9. Nagpur 10. Bhandara 11. Gondiya 12. Gadchiroli 13. Chandrapur 14. Hingoli 15. Parbhani 16. Jalna 17. Aurangabad 18. Nashik 19. Raigarh 20. Pune 21. Ahmadnagar 22. Bid 23. Latur 24. Osmanabad 25. Solapur 26. Satara 27. Ratnagiri 28. Sindhudurg 29. Kolhapur 30. Sangli |
| **Andhra Pradesh-high burden** | 0 | 0 | 0 | 0 | 1. Prakasam 2. Anantpur 3. Chitoor |
| **Karnataka** | 0 | 0 | 0 | 0 | 1. Bagalkot 2. Gulbarga 3. Bidar 4. Raichur 5. Koppal 6. Gadag 7. Dharwad 8. Uttara Kannada 9. Haveri 10. Bellary 11. Shimoga 12. Kolar 13. Mysore 14. Chamarajanagar 15. Yadgir |
| **Goa** | 0 | 0 | 0 | 0 | 1. North Goa 2. South Goa |
| **Kerala** | 1. Idukki 2. Pathanamathitta 3. Thiruvanthapuram | 1. Pathanamthittta | 1. Pathanamthittta | 0 | 1. Mallappuram 2. Palakkad 3. Idukki 4. Pathanamthitta |
| **Tamil Nadu** | 0 | 1. Namakkal 2. Puduukkottai | 1. Thiruvarur 2. Thanjavur 3. Theni | 0 | 1. Thirruvallur 2. Dharmapuri 3. Tiruvannamalai 4. Erode 5. Nilgiris 6. Coimbatore 7. Karur 8. Peramnalur 9. Ariyalur 10. Nagapattinam 11. Sivaganga 12. Madurai 13. Theni 14. Ramanathapuram 15. Thoothukkudi 16. Tirunelveli 17. Krishnagiri 18. Tiruppur |
| **Puducherry** | 0 | 0 | 1. Pondicherry | 0 | 1. Pondicherry 2. Mahe 3. Karaikal |
| **Andaman and Nicobar** | 0 | 1. South Andaman | 0 | 0 | 1. North & Middle Andaman 2. South Andamana 3. Nicobar |
| **Telengana** | 0 | 0 | 0 | 0 | 1. Karimnagar 2. Mahbubnagar 3. Warngal |
